# The Impact of a Standardized Checklist on Transition of Care During Emergency Department Resident Physician Change of Shift

**DOI:** 10.5811/westjem.2018.10.39020

**Published:** 2018-11-20

**Authors:** Alyssa Milano, Holly Stankewicz, Jill Stoltzfus, Philip Salen

**Affiliations:** * St. Luke’s University Health Network, Department of Emergency Medicine, Bethlehem, Pennsylvania; † St. Luke’s University Health Network, Research Institute, Department of Emergency Medicine, Bethlehem, Pennsylvania

## Abstract

**Introduction:**

Transitions of patient care during physicians’ change of shift introduce the potential for critical information to be missed or distorted, resulting in possible morbidity. The Joint Commission, the Accreditation Council for Graduate Medical Education, and the Society of Hospital Medicine jointly encourage a structured format for patient care sign-out. This study’s objective was to examine the impact of a standardized checklist on the quality of emergency medicine (EM) resident physicians’ patient-care transition at shift change.

**Methods:**

Investigators developed a standardized sign-out checklist for EM residents to complete prior to sign out. This checklist included topics of diagnoses, patient-care tasks to do, patient disposition, admission team, and patient code status. Two EM attending physicians, the incoming and departing, assessed the quality of transitions of care at this shift change using a standardized assessment form. This form also assessed overall quality of sign-out using a visual analog scale (VAS), based on a 10-centimeter scale. For two months, we collected initial, status quo data (pre-checklist [PCL] cohort) followed by two months of residents using the checklist (post-checklist [CL] cohort).

**Results:**

We collected data for 77 days (July 1, 2015 – November 11, 2015), 38 days of status quo sign-out followed by 39 days of checklist utilization, comprised of 1,245 attending assessments. Global assessment of sign-out for the CL was 8 compared to 7.5 for the PCL. Aspects of transition of care that implementation of the sign-out checklist impacted included the following (reported as a frequency): “To Do” (PCL 84.3%, CL 97.8%); “Disposition” (PCL 97.2%, CL 99.4%); “Admit Team” (67.1%, CL 76.2%); and “Attending Add” (PCL 23.4%, CL 11.3%).

**Conclusion:**

Implementation of a sign-out checklist enhanced EM resident physician transition of care at shift end by increasing the frequency of discussion of critical tasks remaining for patient care, disposition status, and subjective assessment of quality of sign-out.

## INTRODUCTION

The process of communicating high-quality patient information from one clinician to the next represents one of the ongoing challenges in healthcare.^1^ Providing continuous, round-the-clock patient care mandates effective, succinct, and informative communication between healthcare providers during change of shift.^2^ These clinical handoffs, also known as sign-outs, shift reports, or handovers, occur between multiple providers with various clinical responsibilities throughout the healthcare system. Sign-outs, which are often complex and multifaceted communications, mark the beginning or end of clinical shifts and patient care duties.^3^ They mark the transmission of professional accountability for some, or all, aspects of patient care from one clinician or clinical team to another.^4^ If done poorly, sign-outs can have deleterious clinical impact. The Institute of Medicine attributes a substantial proportion of preventable adverse events to communication errors during sign-out.^5^ These errors are among the root causes of nearly two thirds of potentially significant, preventable adverse clinical outcomes in hospitals.^6^,^7^

Only recently has data become available that demonstrate improvements in sign-outs reduce the rate of subsequent clinical care error.^8^ Substantial variability exists across, and sometimes within, institutions regarding preferred formats and processes for verbal and written handoffs. Research of residency training programs indicates that handoff standardization has not been aggressively implemented or has been implemented with variable compliance.^2^,^9^ The Joint Commission, the Accreditation Council for Graduate Medical Education, and the Society of Hospital Medicine jointly encourage compliance with a structured format for verbally communicating sign-out information.^10^

## OBJECTIVE

Our primary objective was to determine the impact of a standardized checklist on the quality of emergency medicine (EM) resident physicians’ patient care transition at shift change. Secondary objectives included evaluation of the level of EM resident training on perceived quality of transition of care and whether utilization of a sign-out checklist impacted sign-out duration.

## METHODS

After institutional review board (IRB) approval, this single-center prospective study was conducted at an EM residency-affiliated, Level I trauma center emergency department (ED) in Northeastern Pennsylvania. We collected data from July 1, 2015 – November 11, 2015. The cohort consisted of a consecutive sample of EM resident sign-out sessions of all patients present at the time of transfer of care. The departing residents transfer responsibility of all ED patients to the oncoming team under the oversight of attending physicians at the 7 a.m. change of shift. At our institution, morning sign-out is the only time when there is a complete change of shift with attending and resident physicians. Throughout the remainder of the day, there is overlap in the treatment team.

**Figure 1:**
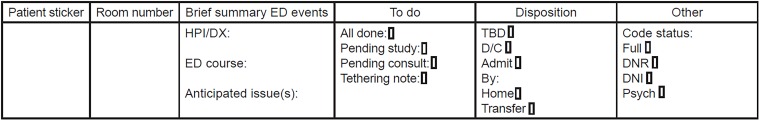
Sign out checklist. *ED*, emergency department; *HPI*, history of present illness; *DX*, diagnoses; Psych, psychiatric; *TBD*, to be determined; *D/C*, discharge; *DNR*, do not resuscitate; *DNI*, do not intubate.

Investigators developed a standardized sign-out method and checklist based on literature review, departmental meetings, and the Joint Commission’s recommended handover communication mnemonic in order to identify key aspects of transition of patient care sign-out (Figure 1).^11^–^13^ Essential aspects of sign-out included the following: diagnoses; patient care tasks to do; patient disposition; and admission team and patient code status. Necessity for patient care clarification from the attending was also noted.

**Population Health Research Capsule**

What do we already know about this issue?

*Transitions of patient care introduce the potential for critical information to be missed or distorted, resulting in possible patient morbidity*.

What was the research question?

Does a standardized checklist impact the quality of Emergency Medicine (EM)resident physicians’ transition of patient care?

What was the major finding of the study?

*Use of a sign out checklist enhanced EM resident physician transition of care*.

How does this improve population health?

*By enhancing the quality of resident physician sign out, there is the potential to limit miscommunication between providers which ultimately can affect patient care and safety*.

**Figure 2:**
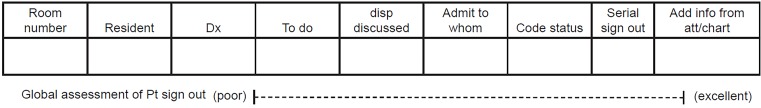
Attending physician scorecard. *DX*, diagnoses; *disp*, disposition; *Psych*, psychiatric; *med*, medical; *att*, attending; *Pt*, patient.

Two EM attending physicians, the incoming and departing, independently assessed the quality of transitions of care at this shift change using a standardized assessment form (Figure 2). This form assessed overall quality of sign-out using a visual analog scale (VAS), which is a 10 centimeter (cm) scale ranging from poor (1cm) to excellent (10 cm). We then evaluated VAS scores overall, as well as a subgroup for each post-graduate year (PGY). Attendings also documented discussion of essential aspects of transition of care issues: primary ED diagnoses; “To Do” (essential tasks to complete); “Disposition” (awaiting discharge, evaluation ongoing, or admission); “Admit Team” (admission service for patient); “Code Status” (regarding living will statements); and “Attending Add” (whether the nocturnal attending needed to clarify the sign-out). A checkmark indicated the topic was mentioned, while a cross-hatched zero indicated it was not discussed.

Assessment of status quo sign-out occurred for 38 days to establish baseline practice patterns, the pre-checklist (PCL) cohort. Resident and attending physicians were given verbal instructions on how to complete the checklist prior to initiation of its use. The following 39 days, residents completed a sign-out study checklist (Figure 1) prior to giving sign out (post-checklist [CL] cohort) to aid in the transition-of-care process.

Time duration of sign-out was also recorded by the attending physicians, defined as the time from first patient sign out to last. Time for the resident to complete the checklist was not included in this measure, as residents were still actively involved in patient care and completed the checklist between their regular shift duties.

### Data Analysis

We completed statistical analysis using SPSS version 25 (Armonk, NY: IBM Corp.). Non-normally distributed continuous data were reported as medians and ranges, with separate Wilcoxon signed rank tests conducted as appropriate. Categorical data were reported as frequencies and percentages. For all analyses, p < 0.05 denotes statistical significance, with no adjustment for multiple testing.

## RESULTS

Assessment of ED departmental resident sign-out occurred for 77 days, 38 days of unstructured, status quo sign-out, and 39 days of using a sign-out checklist. A total of 18 attending physicians, including two nocturnists and 16 rotating day-shift physicians, and 40 resident physicians participated. The total number of days remained consistent before and after initiation of the checklist; however, due to ED census a greater number of assessments was completed in the CL cohort. Table 1 lists a summary of the results with a comparison between the two cohorts, pre- and post-sign out checklist.

Attending physicians completed a total of 1,245 sign-out assessments of EM residents: 548 attending VAS assessments in the PCL and 697 assessments in the CL cohort. Global assessment of transition of care for the CL was 8 (range 2.5 to 10) compared to 7.5 for the PCL (range 0.5 to 9.5) (p < .0001). Aspects of transition of care that implementation of the sign-out checklist impacted included the following (reported as frequencies and percentages): “To Do” (PCL 578/686, 84.3%; CL 482/493, 97.8%; p < 0.0001); “Disposition” (PCL 683/703, 97.2%; CL 518/521, 99.4%; p = 0.004); “Admit Team” (PCL 392/584, 67.1%; CL 321/421, 76.2%; p = 0.03); and “Attending Add” (PCL 100/427, 23.4%; CL 39/345, 11.3%; p < 0.0001). Issues not impacted by the sign-out checklist included “Diagnosis” (PCL 714/727, 98.2%; CL 522/527, 99.1%; p = 0.1) and “Code Status” (PCL 45/505, 8.9%; CL 52/357, 14.6%; p = 0.13).

VAS scores for each PGY are reported at medians and ranges. VAS scores for PGY-1 in PCL cohort was 7.0 (4.0–8.75) and CL 8.0 (6.0–9.5). For PGY-2, PCL cohort median VAS scores were 7.25 (2.25–9.50) and CL cohort 8.5 (4.75–9.75). Lastly, for PGY-3 median VAS PCL was 7.25 (0.75–9.0) and CL 8.0 (4.25–10.0). Results are summarized in Table 2.

Use of a sign-out checklist significantly decreased duration of sign-out by nine minutes mean for the CL cohort, compared to the PCL cohort (P < 0.03).

## DISCUSSION

Prior research has demonstrated that omission or distortion of important clinical information occurs during transition of care.^5^,^6^,^9^,^11^ This study sought to address issues of miscommunication during sign-out through utilization of a standardized checklist. By using a shared departmental model, such as a sign-out checklist, situational awareness of complex information during handoffs is enhanced.^11^ Compared to the status quo unstructured sign-out, implementation of a standardized checklist improved attendings’ perception of the quality of resident transition of care (P < 0.001), discussion of patient care tasks requiring completion (P < 0.001), disposition confirmation (P < 0.004), necessity for attending clarification (P < 0.001), and shorter duration of sign-out process (P< 0.03).

Our checklist did not impact discussion of the working diagnosis and code status. The patient’s working diagnosis is typically one of the first items discussed during sign-outs, which is why we believe this did not improve with implementation of the checklist. In this study a standardized checklist was used; however, during the study time period we found that not all topics, such as code status, were applicable to every patient. All patients present in the ED were signed out during morning sign-out, regardless of acuity. For patients with a lower acuity, it can be suggested that code status does not need to be discussed with patients and members of their treatment team, which likely explains the findings in this study.

Other studies have proposed methods to improve patient transition of care. These include various mnemonics, such as I-PASS (Illness severity; Patient summary; Action list; Situational awareness and contingency plan; Synthesis by receiver) and SIGNOUT (Sick; Identifying data; General course; New events of the day; Overall current clinical status; Upcoming possibilities with plan; Tasks to complete after handoff), which have been evaluated in the inpatient setting. These studies created curriculums and workshops to focus on improving communication during sign out.^12^–^14^ The American College of Emergency Physicians (ACEP) also created a “Safer Sign Out Protocol,” which included the five key components of record, review, round, relay information to the team, and receive feedback.^15^ ACEP has recently provided a similar sign-out checklist; however, we chose a tool that was more tailored to our ED.

Dubosh et al. used an electronic sign-out checklist to evaluate resident sign-out in the ED and found improvements in sign-out without increasing length of time to complete handoff.^16^ Although their study was conducted in the ED, they included residents from various medical specialties and only those in their first and second year of training, unlike our study, which included only EM residents.

Overall, there was slight improvement in each PGY’s VAS assessment following implementation of the checklist. PGY-2 gained the largest improvement, while the residents in their final year of training gained the least improvement. The improvement among first-year residents may have been limited due to the study being conducted during their first few months of residency, when the learning curve is immense in all areas of training. Overall, interns see fewer patients than senior residents and therefore have fewer VAS scores, which can impact their final average. Also, we found that residents in their final year of training were less open to changing how they completed sign-out. Rayo et al. also demonstrated similar findings in their study, which evaluated various team members during sign-out in the critical care setting. Rayo et al. reported that practitioners with higher levels of training provided fewer interjections and a higher proportion of interactive questions.^10^

This study demonstrates that using a sign-out checklist decreases time to complete transition of care. These findings are both statistically and clinically significant, as most providers would like to leave at the end of their shifts. Although the time it took for residents to complete the checklist was not recorded, overall group involvement between the attending and residents was shorter.

## LIMITATIONS

Due to the lack of blinding in the study design, observer bias may have influenced the attending physicians’ assessments of quality, or perceived lack of quality, during the observational and intervention phases of the trial. The Hawthorne effect may have altered the quality of sign-out, as awareness of the trial may have influenced the quality of the subjects’ transition-of-care efforts. Furthermore, participants received a brief verbal instruction on how to complete the checklist and attending scorecard prior to their first time using it. Since a formal group introduction was not given, there may have been inconsistency among the instructions. Although subjects were not randomized into checklist vs. no-checklist cohorts, use of consecutive data of all residents present in the department over three months of data collection controlled for sampling bias.

Attrition bias may have impacted results, as 482/1,245 assessments were incomplete; the most common incomplete assessments were the code status assessments (though this would not apply to all patients) and attending clarifications of clinical information. In addition, due to ED census during the study period, there was a greater number of assessments in the CL cohort. Finally, although implementation of a sign-out checklist resulted in significantly decreased sign-out duration, investigators did not monitor clinician time to complete the checklist. Due to the fact that this was the first study evaluating use of our ED sign-out checklist, further studies will need to be conducted to validate its accuracy and use.

## CONCLUSION

Implementation of a sign-out checklist enhanced EM resident physician transition of care at shift end by increasing the frequency of discussion of critical tasks remaining for patient care, disposition status, and subjective assessment of quality of sign-out, while concurrently decreasing duration of sign-out discussion.

## Figures and Tables

**Table 1:** Impact of a sign-out checklist on outcomes regarding transitions of care at shift end.

	Pre-checklist	Post-checklist	P value
Attending assessment of sign out VAS	7.5 (range 0.5 to 9.5) (n = 548)	8.0 (range 2.5 to 10) (n = 697)	< .0001
+ Diagnosis	714/727, 98.2%	522/527, 99.1%	0.1
− Diagnosis	12/727, 1.7%	5/527, 0.9%	
+ “To do”	578/686, 84.3%	482/493, 97.8%	< .0001
− “To do”	60/686, 8.7%	8/493, 1.6%	
+ Disposition	683/703, 97.2%	518/521, 99.4%	<0.004
− Disposition	14/703, 2%	3/521, .6%	
+ Admit to	392/584, 67.1%	321/421, 76.2%	<0.03
− Admit to	83/584, 14.2%	35/421, 8.3%	
+ Code status	45/505, 8.9%	52/357, 14.6%	0.13
− Code status	295/505, 58.4%	187/357, 52.4%	
+ Attending add	100/427, 23.4%	39/345, 11.3%	<.0001
− Attending add	327/427, 76.6%	306/345, 88.7%	
Sign out duration	13 minutes (mean)	9 minutes (mean)	< 0.03

(+) topic mentioned by resident; (−) topic omitted by resident.

*VAS*, visual analog scale.

**Table 2:** Visual analong scale (VAS) assessments of sign-out quality.

	VAS PCL	VAS CL
PGY-1	7.0 (4.0–8.75)	8 (6.0–9.5)
PGY-2	7.25 (2.25–9.5)	8.5 (4.75–9.75)
PGY-3	7.25 (0.75–9)	8.0 (4.25–10.0)

*PGY*, post-graduate year; *PCL*, pre-checklist; *CL*, post-checklist.
